# MDA-DETR: Enhancing Offending Animal Detection with Multi-Channel Attention and Multi-Scale Feature Aggregation

**DOI:** 10.3390/ani15020259

**Published:** 2025-01-17

**Authors:** Haiyan Zhang, Huiqi Li, Guodong Sun, Feng Yang

**Affiliations:** 1 School of Information Science and Technology, Beijing Forestry University, Beijing 100083, China; zhyzml@bjfu.edu.cn (H.Z.); shangguanli21@bjfu.edu.cn (H.L.); sungd@bjfu.edu.cn (G.S.); 2 Engineering Research Center for Forestry-Oriented Intelligent Information Processing of National Forestry and Grassland Administration, Beijing 100083, China

**Keywords:** object detection, RT-DETR, transformer, computer vision, attention mechanism

## Abstract

Conflicts between humans and animals in agricultural and settlement areas have recently increased, resulting in significant resource loss and risks to human and animal lives. This growing issue presents a global challenge. This paper addresses the detection and identification of offending animals, particularly in obscured or blurry nighttime images. This article introduces Multi-Channel Coordinated Attention and Multi-Dimension Feature Aggregation (MDA-DETR). It integrates multi-scale features for enhanced detection accuracy, employing a Multi-Channel Coordinated Attention (MCCA) mechanism to incorporate location, semantic, and long-range dependency information and a Multi-Dimension Feature Aggregation Module (DFAM) for cross-scale feature aggregation. Additionally, the VariFocal Loss function is utilized to assign pixel weights, enhancing detail focus and maintaining accuracy. In the dataset section, this article uses a dataset from the Northeast China Tiger and Leopard National Park, which includes images of six common offending animal species. In the comprehensive experiments on the dataset, the $mAP_{50}$ index of MDA-DETR was 1.3%, 0.6%, 0.3%, 3%, 1.1%, and 0.5% higher than RT-DETR-r18, yolov8n, yolov9-C, DETR, Deformable-detr, and DCA-yolov8, respectively, indicating that MDA-DETR is superior to other advanced methods.

## Simple Summary

As agriculture and human settlements expand, conflicts between humans and animals become more frequent, resulting in resource loss and safety risks. Therefore, accurately identifying and locating offending animals is essential. This study presents a novel method for automatically detecting offending animals, especially in situations where they are obscured or the images are unclear. This research focused on six types of offending animals commonly found in northeastern China. By employing improved image processing techniques, this study enhanced detection accuracy in complex environments. Experimental results demonstrate that the proposed method outperforms existing techniques on the dataset used. This new approach helps improve the accuracy of intelligent monitoring systems, providing better technical support to minimize conflicts between humans and animals, thus protecting agriculture and ensuring the safety of both humans and animals.

## 1. Introduction

With the rapid advancement of urbanization, human activities are increasingly encroaching on wildlife habitats. This has led to a rise in harmful wildlife incidents, despite efforts to establish national parks and protected areas. Such incidents not only pose a threat to human health and livelihoods but also highlight the urgent need to address wildlife–human conflicts [1]. As a result, mitigating these conflicts has become a global priority for governments and a focus of academic research.

Traditional methods for managing wildlife–human conflicts, such as erecting physical barriers like barbed wire or electric fences around settlements and agricultural areas, have notable limitations. These measures can induce stress responses or even fatalities in animals and pose potential risks to humans [2]. To overcome these challenges, there is a growing demand for intelligent surveillance systems capable of automatically detecting and identifying offending animals in real time.

Object detection, a fundamental yet challenging task in computer vision, involves assigning precise bounding boxes and classification labels to objects in images. Recent advances in deep learning, coupled with reduced hardware costs, have spurred the development of robust object detection algorithms. Two-stage detection frameworks such as Faster R-CNN [3,4] and R-CNN [5], as well as single-stage detectors like YOLO [6,7,8,9], have gained prominence. Moreover, the dominance of Convolutional Neural Networks (CNNs) in this field has recently been challenged by the rise of Transformers, which leverage attention mechanisms to boost model performance. DETR, the first Transformer-based object detection model [10], eliminates the need for manually designed anchor frames and non-maximal suppression (NMS). However, it faces limitations in processing speed. In 2023, Baidu’s Flying Paddle team introduced RT-DETR, a breakthrough in object detection. This model transitions from “dense detection” to “sparse detection”, eliminating threshold filtering and NMS. It delivers an end-to-end, real-time object detection solution [11], making it a promising tool for applications like offending animal detection.

Offending animals frequently appear near planting and breeding areas, causing damage to vegetation and livestock. These activities often obscure the animals within the damaged vegetation. Furthermore, many offending animals are nocturnal, leading to blurry images captured by trap cameras. This study emphasizes the crucial need for accurate detection and recognition of obscured or blurry images of offending animals. While the RT-DETR model exhibits strong robustness and generalization, it has limitations. The AIFI module, relying solely on a self-attention mechanism, provides rich semantic information but lacks precise positional information. Additionally, the CCFF module, despite integrating cross-scale information, struggles with detecting targets in blurry images.

To address these limitations, this paper proposes MDA-DETR, a novel method based on Multi-Channel Coordinated Attention and multi-scale feature aggregation, to enhance the detection of obscured and blurry images of offending animals. Inspired by channel attention mechanisms [12], we introduce a Multi-Channel Coordinated Attention (MCCA) mechanism. This mechanism extracts image features along three spatial directions, capturing long-range dependencies and providing precise positional information while preserving the rich semantic information from deep networks. This significantly improves the accuracy of detecting occluded targets.

Furthermore, inspired by the RepBiPAN module [13], we propose a Multi-Dimension Feature Aggregation Module (DFAM). This module effectively fuses positional information from shallow networks with semantic information from deeper networks, and aggregates features from multiple scales. This enhances the detection accuracy of blurry images. To reduce the overall parameter count, we incorporate the RepNCSPELAN4 module [14] into the backbone network.

MDA-DETR was rigorously evaluated on a subset of the Northeast China Tiger and Leopard National Park dataset [15]. Ablation experiments were conducted to assess the performance of each component, demonstrating the superior performance of MDA-DETR in detecting offending animals. The main contributions of this study are as follows:

(i) **Multi-Channel Coordinate Attention (MCCA) mechanism** This mechanism extracts image features along three spatial directions, integrating precise positional and long-range dependency information while preserving the rich semantic information from deep networks.
(ii) **Multi-Dimension Feature Aggregation Module (DFAM)** This module effectively fuses multi-scale feature maps, enabling the extraction of complementary and global information.
(iii) **Comparative experiments** demonstrate the superiority of MDA-DETR over state-of-the-art methods. Ablation experiments confirm the effectiveness of each key component.

## 2. Related Works

### 2.1. Transformer-Based Object Detection

Transformer is an encoder–decoder architecture based on a self-attention mechanism, first proposed by Google and applied to Natural Language Processing (NLP), achieving state-of-the-art (SOTA) results in various NLP tasks [16]. In 2020, Nicolas Carion et al. introduced Transformer to object detection with the DETR (DEtection TRansformer) model, simplifying the detection process by treating it as an ensemble prediction problem and eliminating the need for manually designing components. In addition, DETR converts the feature image output from the backbone network into a one-dimensional sequence, enabling the model to compute correlations between each pixel and others, thus achieving a wider receptive field than that of CNNs [17].

However, as a pioneering work, DETR has some limitations. Zhu et al. proposed Deformable-detr [18] to address the slow training speed of Transformer in computer vision. Its attentional module focuses only on the key sampling points, resulting in better performance on small targets and reduced training time. In 2022, Wang et al. introduced Anchor DETR [19], a new Transformer-based query mechanism for object detection. This model employs a new attentional variant to predict multiple objects in a region, thus effectively solving the problem of ‘one region, multiple objects’.

Although the Transformer-based DETR series has challenged the monopoly of CNN in just two years, it is still no substitute for the CNN-based series of algorithms in terms of real-time performance in industrial applications. To address this, Baidu’s team, composed of Lv et al., proposed RT-DETR (Real-Time DEtection Transformer) in 2023 [11]. It is a real-time end-to-end detector based on the DETR model and outperforms YOLO in real-time object detection. Compared with yolov8, RT-DETR requires a shorter training duration, fewer data enhancement strategies, and demonstrates stronger performance under the same test conditions.

Most state-of-the-art Transformer methods divide images into regular grids and represent each grid region with visual tokens. However, the fixed token distribution ignores the semantic information of different areas of the image, which leads to performance degradation. To address this issue, in 2024, Wang Zeng et al. proposed the Token Clustering Transformer (TCFormer) [20]. This method dynamically generates visual tokens based on semantic information, allowing regions with similar semantics to be represented by the same token, even if these regions are not adjacent. For areas containing important details, TCFormer uses fine-grained tokens to enhance the accuracy of image understanding. In the same year, Yansong Peng et al. redefined the bounding box regression task in the DETR model and proposed the D-FINE model [21]. This model introduces Fine-grained Distribution Refinement (FDR) and Global Optimal Localization Self-Distillation (GO-LSD) strategies, transforming the regression process from predicting fixed coordinates to iteratively refining probability distributions, thus achieving higher accuracy in object localization.

### 2.2. Wildlife Object Detection

Wildlife surveys are key to nature conservation. In 2021, Delplanque et al. evaluated the performance of three CNN algorithms: Faster-RCNN, Libra-RCNN [22], and RetinaNet [23]. They trained the models using an independent dataset, enabling them to detect and identify African mammal species based on high-resolution aerial imagery. The Libra-RCNN model [24] with the best detection results accurately detected animals in open and sparse grasslands, reducing detection time. In 2022, Cai Qianzhou et al. proposed a solution [25] for long-tailed data based on YOLOv4-Tiny. Their approach combined two-stage learning and reweighting. In the first stage, the model was trained without weighting. In the second stage, weights from the first stage were used in combination with the reweighting method, ultimately improving the model’s accuracy for long-tailed data acquired by trap cameras. In 2023, Roy et al. proposed the WilDect-YOLO model [26] for the automatic detection of endangered wildlife. This model introduces residual blocks in the backbone network and integrates DenseNet blocks to extract and retain critical feature information. Additionally, Spatial Pyramid Pooling (SPP) and an improved Path Aggregation Network (PANet) are added to the feature fusion part, enhancing the model’s perceptual field and preserving fine-grained local information. In 2024, Yang Wenhan et al. combined the Swin Transformer module with a CNN to propose a method [27] for detecting wildlife images captured by trap cameras based on YOLOV5s. The technique fuses the advantages of both networks in the feature extraction layer, expanding the receptive field of feature extraction and enabling the model to accurately detect animals despite severe occlusion, low contrast with the background, and other challenges.

### 2.3. Offending Animal Detection

Timely detection and identification of offending animals are crucial to reducing human–animal conflicts. In 2018, Ram et al. proposed an automatic unsupervised elephant image detection system [28] for human–elephant conflicts. This system acquires animal images and presence signals from cameras and sensors, respectively. Placed near conflict-prone areas in forest villages, the device generates a warning signal when an elephant is detected, and then sent to a specific location via a GSM module. In 2020, Ravoor et al. designed a cross-camera tracking system [29] for detecting jaguars, elephants, and other offending animals. The method uses the MobileNetv2-SSD [30] model to localize the animals and the Triplet Loss trained ResNet-50 [31] model for re-identification. The integrated model runs at 2–3 frames per second, enabling near real-time functionality. In 2022, Lee et al. proposed an extract–append data enhancement method [32] that extracts specific objects from a limited number of images through semantic segmentation and appends them to numerous images with arbitrary backgrounds. The technique generates images of offending animals with varied backgrounds, improving the model’s detection performance by enriching the dataset. In 2023, Charles et al. developed a system [33] consisting of an Arduino, a PIR motion sensor, an LED flash, a speaker, and an acoustic cannon to monitor a field 24×7 h a day. When the PIR motion sensor detects an animal, the Arduino activates, the speaker emits a threatening animal sound, the LED light flashes, and the system sends a text message and a photo to the farmer within seconds of detection.

## 3. Materials and Methods

### 3.1. Dataset

This study uses the publicly available Northeast China Tiger and Leopard National Park dataset [15]. From this dataset, six common species of offending animals in northeastern China—badger, black bear, leopard cat, red fox, weasel, and wild boar—were selected. The total dataset comprises 9641 images, including 5823 daytime images and 3818 nighttime images. The dataset was divided into training, validation, and test sets in a 7:2:1 ratio. Mosaic data augmentation was applied to the training set to enhance the dataset (see Table 1).

### 3.2. Overall Architecture

**Figure 1:**
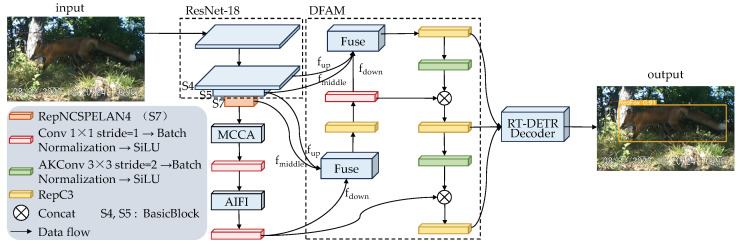
The overall structure of MDA-DETR.

The overall structure of MDA-DETR proposed in this paper is shown in Figure 1, which is a Transformer-based encoding–decoding structure. First, the improved ResNet-18 backbone network is used for encoding and extracting image features. This paper acquires three different scales of features with the resolutions: S4[M,N], $S5[\frac{M}{2},\frac{N}{2}]$, $S7[\frac{M}{4},\frac{N}{4}]$. Then, the S7 deep feature images are inputted into MCCA to obtain accurate position information. In addition, the AIFI self-attention mechanism in the RT-DETR model is retained to extract rich semantic information. Next, the feature maps of three different scales—shallow, intermediate, and deep—are further fused by DFAM to obtain a new feature image with global information. Finally, the Variable Focal Loss function (VariFocal Loss) [34], GIoU loss function [35], and L1-loss [36] are used to measure the error between the model’s prediction results and the actual labels and to adjust the weight parameters accordingly to optimize the overall model further.

### 3.3. Multi-Channel Coordinate Attention (MCCA)

**Figure 2:**
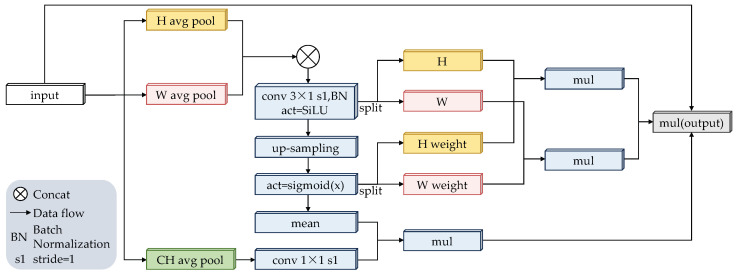
The structure diagram of MCCA.

Generally, channel attention mechanisms play a crucial role in improving model performance. However, traditional channel attention mechanisms focus primarily on extracting semantic information from feature maps, often neglecting the importance of target position [37]. In the field of detecting offending animals, obstructions often occur, making accurate positional information critical for enhancing model predictive performance. Inspired by channel attention mechanisms, this paper proposes the Multi-Channel Coordinated Attention Mechanism (MCCA). It effectively extracts semantic and positional information from feature maps through three spatial directions and captures long-range dependencies (as shown in Figure 2), ultimately improving the accuracy of detecting obscured offending animals.

Specifically, the input image of size M×N is first decomposed into three one-dimensional feature encoding processes. The three outputs can be expressed as follows:

(1) $$\left\{\begin{array}{c} Z\left(h\right)=\frac{1}{W}\sum_{j=1}^{W}x\left(h,j\right)\\ Z\left(w\right)=\frac{1}{H}\sum_{i=1}^{H}x\left(i,w\right)\\ Z\left(ch\right)=\sum_{i=1}^{H}\sum_{j=1}^{W}x\left(i,j\right) \end{array}\right.$$

The features of *H* and *W* are then combined to obtain $Z_{concat}=\left[Z\left(h\right),Z\left(w\right)\right]$. These combined features are then fed into a 3×1 convolution kernel for convolution, followed by batch normalization and SiLU activation [38]. This process can be described as follows:

(2) $$F_{1}=B_{conv}\left(Z_{concat}\right)$$

where $B_{conv}\left(\cdot \right)$ represents a series of operations, including 3×1 convolution, batch normalization, and SiLU activation functions.

Subsequently, $F_{1}$ is decomposed into two separate tensors along the *H* and *W* directions to obtain $F_{1}^{h}$ and $F_{1}^{w}$. Upsampling and sigmoid(x) activation operations are then performed on $F_{1}$. The sigmoid(x) activation function is used here to reduce the complexity and computational overhead of the module. This process can be described as follows:

(3) $$F_{2}=sigmoid\left(U\left(F_{1}\right)\right)$$

where U(·) denotes upsampling.

Decomposing $F_{2}$ into two separate attentional weights along the *H* and *W* directions yields $F_{2}^{h}$ and $F_{2}^{w}$ for subsequent refinement of the $F_{1}^{h}$ and $F_{1}^{w}$ tensors. Next, $F_{2}$ is batch normalized. Afterward, it is multiplied with Z(ch), which has been processed through a 1×1 convolution, to obtain $F_{3}$. This process can be defined as follows:

(4) $$F_{3}=F_{2}\times Conv\left(Z\left(ch\right)\right)$$

The resulting output $F_{out}$ can be written as

(5) $$F_{out}=F_{1}^{h}\times F_{1}^{w}\times F_{2}^{h}\times F_{2}^{w}\times F_{3}$$

### 3.4. Multi-Dimension Feature Aggregation Module (DFAM)

**Figure 3:**
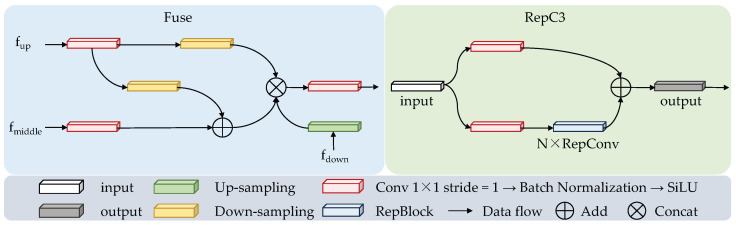
Structure of Fuse and RepC3 in DFAM.

When detecting offending animals, their rapid movements and nighttime appearances can result in blurry images and unclear boundaries when captured by trap cameras. Utilizing the MCCA module and the AIFI module, the model obtains feature images with precise localization and rich semantic information. However, recognition accuracy for such challenging conditions remains an issue. To leverage the complementarity and correlation between multi-scale features, this paper proposes a multi-scale feature aggregation module (DFAM), inspired by the RepBiPAN module, to fuse feature images from the backbone network with those processed through MCCA and AIFI operations. Figure 1 illustrates the overall structure of DFAM. The main contribution of DFAM is the fusion of multiple feature images at different scales, utilizing both element-wise addition and cascading operations. This approach enhances information at the same scale while benefiting from features at other scales. Additionally, this paper introduces an aggregation block (Fuse) that consolidates feature maps of varying sizes in the DFAM fusion path, followed by a convolution block (RepC3) composed of multiple 1×1 convolutional layers. These operations enable the model to integrate feature maps from each layer, yielding feature images with globally essential features and improving the detection accuracy of blurry animal images. Figure 3 shows the structure of Fuse and RepC3 in DFAM.

Specifically, the Fuse module handles three inputs to the aggregation block: $f_{up}$, $f_{middle}$, and $f_{down}$. For the shallow feature map, $f_{up}$ is first processed by a 1×1 convolution, followed by batch normalization and the SiLU activation function, and then undergoes downsampling to facilitate subsequent fusion operations. This process can be expressed as follows:

(6) $$f_{1}=D\left(B_{conv}^{1}\left(f_{up}\right)\right)$$

where $B_{conv}^{1}\left(\cdot \right)$ is a series of sequential operations, including 1×1 convolution, batch normalization, and SiLU activation functions.

After that, the middle layer feature map $f_{middle}$ is input into $B_{conv}^{1}\left(\cdot \right)$ for a convolution operation with a 1×1 kernel, and the deep layer feature map $f_{down}$ is upsampled. This process can be represented as follows:

(7) $$\left\{\begin{array}{cc} f_{2} & =B_{conv}^{1}\left(f_{middle}\right)\\ f_{3} & =U(f_{down}) \end{array}\right.$$

where U(·) denotes upsampling.

Then, $f_{1}$ and $f_{2}$ are fused using element-wise addition, and the fused feature maps are cascaded with $f_{1}$ and $f_{3}$, and finally input into $B_{conv}^{1}\left(\cdot \right)$ for a convolution operation. This process can be defined as follows:

(8) $$f_{out}=B_{conv}^{1}\left(concat\left(\left(f_{1}+f_{2}\right),f_{1},f_{2}\right)\right)$$

In the RepC3 convolutional block, the input features first undergo sequential operations to adjust the number of channels. After this, feature extraction is performed on one of the branches using a RepBlock consisting of N RepConvs [39]. Finally, the outputs of the dual paths are fused using element-wise addition. By employing DFAM, the model can fuse features at three different scales and utilize cross-level features to acquire more global and complementary information. This results in a more expressive fused feature image that accurately detects the presence of offending animals in blurred images.

### 3.5. Backbone Network Improvement Strategy

Although the images processed by MCCA and DFAM retain accurate positional information and rich semantic information, the overall number of model parameters is high. To address this, we use the RepNCSPELAN4 structure to replace the last layer of the ResNet-18 backbone network. The RepNCSPELAN4 structure combines CSPNet with gradient path planning and ELAN, extending ELAN to support GELAN for any computational block. This design is focused on achieving lightweight inference speed and accuracy. This optimization strategy enables the model to reduce the number of parameters while maintaining the original accuracy.

### 3.6. Loss Function

The Variable Focal Loss function (VariFocal Loss) uses the IoU-aware Classification Score (IACS) to represent the probability of target object ranking of candidate detection frames. The loss function employs an asymmetric weighting approach to improve the model’s classification performance by reducing the weight of negative samples and increasing the weight of positive samples. This allows the model to better focus on training with high-quality positive samples. The functional representation of VariFocal Loss is as follows:

(9) $$L_{VFL}=\left\{\begin{array}{cc} -y^{\left(i\right)}\left(y^{\left(i\right)}\log\left({\hat{y}}^{\left(i\right)}\right)+\left(1-y^{\left(i\right)}\right)\log\left(1-{\hat{y}}^{\left(i\right)}\right)\right) & \;y^{\left(i\right)}>0\\ -\alpha {\hat{y}}^{(i)\gamma }\log\left(1-{\hat{y}}^{\left(i\right)}\right) & \;y^{\left(i\right)}=0 \end{array}\right.$$

where $y^{\left(i\right)}$ denotes the label of the sample *i* with positive class 1 and negative class 0. ${\hat{y}}^{\left(i\right)}$ denotes the predicted IACS for the sample *i*. α and ${\hat{y}}^{(i)\gamma }$ represent the scalability coefficients of the moderating loss.

Traditional object detection algorithms typically use the IoU loss function to measure the degree of overlap between the prediction frame and the ground truth. However, when the overlap is high, the IoU loss function suffers from the vanishing gradient problem, making further model optimization difficult [40]. To better localize the target and reduce the absolute error between the prediction and ground truth, the GIoU loss function and L1-loss are introduced. Specifically, the GIoU loss function introduces a penalty term on top of the IoU loss function to measure the non-overlap between the prediction and ground truth frames, addressing the vanishing gradient problem and enabling faster convergence. The L1-loss, or mean absolute error (MAE), is the average of the absolute errors between the model’s predicted and true values. Using both the GIoU loss function and L1-loss provides better supervision during model training and ensures the accuracy of its results.

The expressions for the loss functions are as follows:

(10) $$L_{GIoU}=1-IoU+\frac{|A_{C}-U|}{|A_{C}|}$$

(11) $$L_{1}=\frac{1}{N}\sum_{i=1}^{N}\left|y_{i}-y_{i}^{\prime }\right|$$

where IoU denotes the intersection ratio between the predicted and ground truth boxes, $A_{c}$ represents the minimum bounding box containing both the predicted and ground truth boxes, and *U* represents the union of predicted and real bounding boxes. *N* is the number of samples, $y_{i}$ is the true value of sample *i*, and $y_{i}^{\prime }$ is the predicted value of sample *i*.

Finally, the overall loss function for the proposed model is as follows:

(12) $$L=L_{VFL}+L_{GIoU}+L_{1}$$

## 4. Results

### 4.1. Evaluation Metrics

To evaluate the model proposed in this study, six widely used evaluation metrics in object detection tasks were adopted: Precision, Recall, $mAP_{50}$ (mean Average Precision), *mAP*_50–95_, parameters, and FPS (Frames Per Second). In object detection tasks, the Intersection over Union (IoU) between the predicted bounding box and the ground truth box is generally used to assess their overlap, thereby evaluating the model’s detection accuracy on a specific dataset. A higher IoU value indicates greater similarity between the two boxes. If the IoU exceeds a certain threshold (commonly set at 0.5, also used in this paper), the model is considered to have successfully detected the object, and the result is classified as a positive example (*P*); otherwise, it is treated as a negative example (*N*). For a positive example, if the model’s classification result is correct, it is considered a true positive (TP); otherwise, it is regarded as a false positive (FP). If a ground truth box is not detected, it is labeled as a false negative (FN). Based on these, Precision and Recall can be calculated using the following formulas:

(13) $$\begin{array}{c} Precision=\frac{TP}{TP+FP} \end{array}$$

(14) $$\begin{array}{c} Recall=\frac{TP}{TP+FN} \end{array}$$

However, Precision and Recall do not fully reflect the performance of an object detection model across different thresholds. When Precision increases, Recall often decreases; conversely, when Recall increases, Precision tends to decrease. This is because the stricter the criteria for predicting a sample as a positive example, the more likely the model is to miss some true positive examples. On the other hand, the more lenient the criteria, the more likely the model is to increase the number of false positives. Therefore, this study includes two additional evaluation metrics: $mAP_{50}$ and *mAP*_50–95_. $mAP_{50}$ represents the average precision (AP) across multiple categories at an IoU threshold of 0.5. *mAP*_50–95_ represents the average of mAP results at 10 IoU thresholds ranging from 0.5 to 0.95, with a step size of 0.05. The higher the mAP value, the better the model’s detection performance. The formulas for calculating AP and mAP are as follows:

(15) $$\begin{array}{c} AP=\sum_{k=1}^{N}P\left(s\right)\cdot \Delta R\left(s\right) \end{array}$$

(16) $$\begin{array}{c} mAP=\frac{1}{c}\sum_{i=1}^{c}AP\left(i\right) \end{array}$$

where *P* represents Precision, *R* represents Recall, *s* represents the classification confidence of the detection box, *N* represents the number of discrete points, and *c* represents the number of categories.

For model complexity and detection speed, this study uses two metrics: Frames Per Second (FPS) and parameters. FPS indicates the number of frames the object detection model can process per second. The higher the FPS value, the faster the detection speed and the better the real-time performance of the model. Parameters reflect the spatial complexity of the model. The expression for FPS is as follows:

(17) $$\begin{array}{c} FPS=\frac{1000\;\mathrm{ms}}{preprocess+inference+NMS} \end{array}$$

where 1000 ms refers to 1000 milliseconds, preprocess refers to the time required for the preprocessing stage, inference refers to the time required for the model’s inference, and NMS refers to the post-processing time required when the model applies Non-Maximum Suppression.

### 4.2. Parameter Settings

The overall model is implemented using the Pytorch framework and trained on a Titan RTX3090 GPU. During training, the pre-trained weights of the ResNet-18 [31] on ImageNet-1k [41] were used and trained on the public dataset used in this paper. The model used the AdamW optimizer with a weight decay of 0.0001, an initial learning rate set to 0.0001, and a mosaic data enhancement probability of 0.5.

### 4.3. Comparative Experiments on Backbone Network Improvement Strategies

This paper compares the model with only the backbone network part to the one with the RT-DETR decoder part. The results show that the improved model reduces the number of parameters by 46.75% compared with the original model using the ResNet-18 backbone network. Additionally, it achieves some improvement in accuracy, as shown in Table 2.

### 4.4. Ablation Study

To evaluate the effectiveness of each key component in MDA-DETR, this paper conducted comprehensive ablation experiments focusing on (1) the effectiveness of backbone network improvement strategies, (2) the effectiveness of MCCA, and (3) the importance of DFAM. During these experiments, two challenging scenarios—occlusion and blurring—were selected to observe and record the performance of these key components under different conditions, demonstrating their effectiveness in various situations.

**Figure 4:**
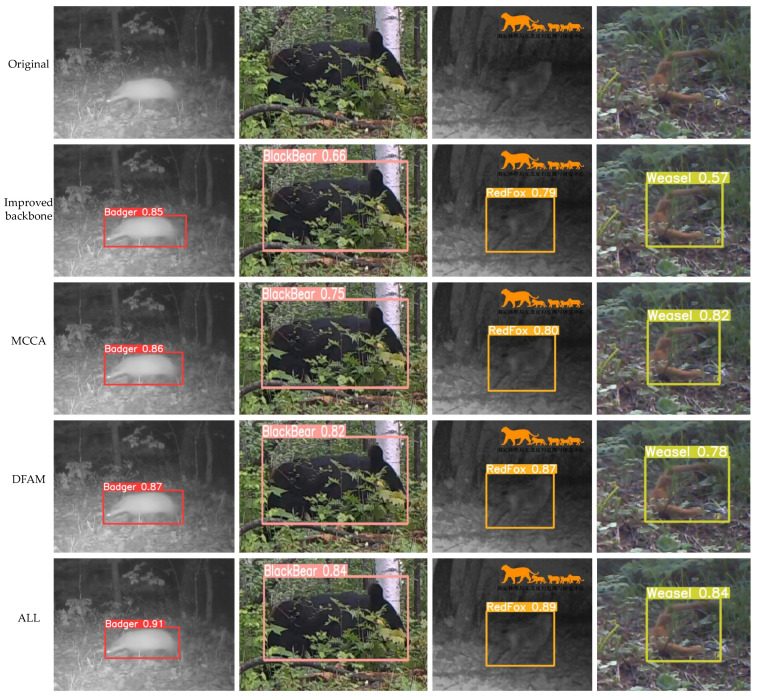
Comparison of ablation experiments. (The orange animal in the upper right corner of the third column is the dataset icon.)

(1) Effectiveness of Backbone Network Improvement Strategies: The backbone network employs the RepNCSPELAN4 structure, initially enhancing the backbone’s performance and shows improvement across four indicators compared with the model with only the ResNet-18 backbone network (as shown in the second row of Table 3). However, when animals are occluded, the detection accuracy is low (as shown in the second row and fourth column of Figure 4).
(2) Effectiveness of MCCA: This study retained only the improved backbone network and MCCA for training and testing to demonstrate its effectiveness. The results indicate that MCCA significantly improves all four evaluation metrics and further enhances the accuracy of detecting occluded offending animals (as shown in the third row and fourth column of Figure 4).
(3) The effectiveness of DFAM. DFAM adjusts the number of channels in the feature map generated by the AIFI module and further fuses it with the feature map generated by the improved backbone network. To demonstrate DFAM’s effectiveness, this study retained only DFAM and the improved backbone network for testing. Table 3 shows that adding DFAM leads to a slight decrease in recall. However, the 0.3% decrease in recall is accompanied by a 1.2% increase in precision, making the slight drop in recall acceptable. Furthermore, Figure 4 shows that using this module has enhanced the accuracy of generating the minimum bounding rectangle. Particularly when the offending animal in the image is blurry, it can more accurately locate and identify the offending animal. Finally, by combining the three proposed improvement methods, the model achieved a 2.9% increase in accuracy, a 3.3% increase in recall, a 2.9% improvement in $mAP_{50}$, and a 4.7% improvement in *mAP*_50–95_ compared with the original baseline. The detection performance was exceptional, proving the effectiveness of the improvement strategy proposed in this article.

### 4.5. Comparison with Other Models

To comprehensively evaluate the performance of MDA-DETR, this paper compares it with six state-of-the-art object detection models: RT-DETR-r18 [11], yolov8n [42], yolov9-C [14], DETR [10], Deformable-detr [18], and DCA-yolov8 [43]. The results for all models were generated using the official code or based on published papers.

Table 4 shows the results of comparing MDA-DETR with six state-of-the-art methods, where the red font and blue font indicate the best and second best performance. From the results in Table 4, it can be seen that the proposed model achieves 97.8% $mAP_{50}$ metrics on the dataset, outperforming the original RT-DETR-r18 model by 1.3%, and it is 0.6% better than yolov8n, 0.3% better than yolov9-C, 3% better than DETR, 1.1% better than Deformable-detr, and 0.5% better than DCA-yolov8. Overall, the model proposed in this article outperforms other advanced methods in the detection of offending animals on the part of the publicly available Northeast China Tiger and Leopard National Park dataset and can better detect images of accident-prone animals.

**Figure 5:**
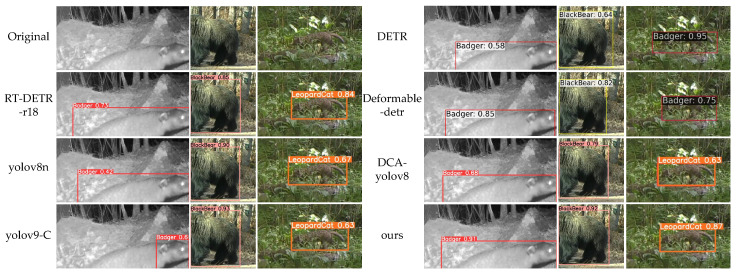
Comparison between ours and advanced methods (badger, black bear, leopard cat).

**Figure 6:**
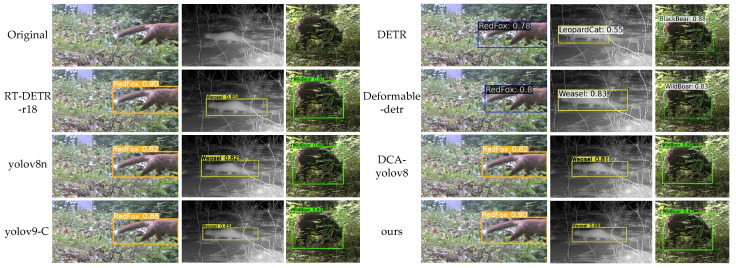
Comparison between ours and advanced methods (red fox, yellow weasel, wild boar).

Figure 5 and Figure 6 show the detection results of MDA-DETR and advanced methods in different scenarios. The images selected in this article include three scenarios: animals being occluded, only having partial animal features, and animals being blurry at night. Among them, DETR and Deformable-detr have better performance in detecting images with only partial animal features, but there are false detections when detecting occluded animal images (leopard cats), and DETR also has false detections when detecting occluded wild boars. Yolov8n and yolov9-C can effectively handle scenes with only partial animal features and animal occlusion, but when detecting blurry animal images at night, yolov8n has lower detection accuracy, while yolov9-C lacks comprehensive detection (badgers). This article selects the DCA-yolov8 model, which can also detect occluded scenes, as the comparison model. From the results, it can be seen that the MDA-DETR proposed in this article has higher detection accuracy than DCA-ylov8 in several complex tasks. Overall, the method proposed in this article outperforms other methods on the publicly available dataset used. MDA-DETR not only accurately classifies the offending animal but also accurately locates and detects the offending animal in scenes where the animal is occluded and blurred at night.

## 5. Discussion

This study proposed MDA-DETR, a novel object detection model, to address the challenges of detecting offending animals, particularly in obscured and blurry nighttime images. Comprehensive evaluations on the Northeast China Tiger and Leopard National Park dataset demonstrated that MDA-DETR significantly outperforms six state-of-the-art models, achieving superior results in mAP50 (+1.3% to +3%) and mAP50-95 (+0.5% to +4.7%). These results validate the effectiveness of the Multi-Channel Coordinated Attention (MCCA) mechanism and the Multi-Dimension Feature Aggregation Module (DFAM) in improving detection accuracy under challenging conditions.

The main findings of this research align with the initial hypothesis that incorporating multi-scale feature aggregation and enhanced attention mechanisms would address limitations in existing models. The observed improvements can be attributed to the following factors: The MCCA mechanism effectively integrates spatial and long-range dependencies, enhancing the accuracy of detecting occluded animals. The DFAM module fuses features across multiple scales, providing global and complementary information to improve the detection of blurry images. The use of VariFocal Loss further enhances classification accuracy by emphasizing high-quality positive samples.

Compared with the YOLO series, DETR, and its variants, MDA-DETR demonstrates significant advantages in handling occlusion and nighttime detection on the dataset used in this study. While the existing YOLO and DETR series methods perform well in most conventional object detection tasks, YOLO models tend to lose crucial information when processing blurred images of animals at night, leading to inaccurate target localization. Although DETR and its variants have improved target detection localization, they often fail to correctly classify objects in complex backgrounds, especially in occlusion or cluttered backgrounds. In contrast, MDA-DETR effectively overcomes these challenges by combining MCCA and DFAM. By incorporating multi-scale feature fusion and integrating the image’s positional information, semantic information, and long-range dependencies, MDA-DETR avoids detection failures caused by the loss of local features. Additionally, this multi-level information integration enhances the model’s ability to detect occluded animals and blurred nighttime animal images, reducing false negatives and false positives, and providing stronger robustness.

Despite the significant advantages demonstrated by MDA-DETR, this study still has certain limitations. First, the dataset used in this study contains only six specific species of offending animals, which limits the model’s applicability to other species and broader scenarios. Second, the dataset does not cover more complex environmental factors, such as changing lighting conditions or motion blur caused by rapid movement, which can often affect the model’s performance in detection tasks. Furthermore, although MDA-DETR has achieved significant accuracy improvements, its computational complexity may pose challenges for real-time deployment on edge devices.

To address these limitations, future research will focus on the following areas: Expanding the dataset: Plans are in place to create a dataset that includes more animal species and more complex environmental factors. Additionally, the use of generative models or trap cameras will be considered to acquire the relevant images for further evaluating the model’s performance in various scenarios, thereby enhancing its generalization ability and applicability to a broader range of species. Optimizing the model architecture: considering the computational complexity involved in real-time deployment, future work will explore lighter model architectures, such as integrating more efficient attention mechanisms or designing low-computation overhead feature extraction modules, to better suit resource-constrained edge devices. Expanding the application scope: Future studies will further explore the potential of MDA-DETR in a wider range of application scenarios, including general wildlife detection and endangered species protection. By expanding its application scope, this study wishes MDA-DETR to play a greater role in protecting ecological environments and biodiversity.

## 6. Conclusions

We propose a novel model, MDA-DETR, for detecting offending animals. To better handle scenes where animals are occluded, the MCCA module is designed to extract semantic information from feature maps, target location information, and long-distance dependencies. Subsequently, the DFAM module aggregates features at three different scales to obtain global features, enhancing the accuracy of detecting offending animals in blurry images. The data used in this study come from the Northeast Tiger and Leopard National Park dataset. Ablation experiments on this dataset demonstrate the effectiveness of the key components in MDA-DETR. Additionally, comprehensive comparative experimental results on this dataset confirm the robustness and effectiveness of the MDA-DETR model.

## Figures and Tables

**Table 1:** Brief information about the dataset used in this article.

Animal	Total Number of Images	Number of Nighttime Images	Number of Daytime Images	Number of Instances
Badger	1309	921	388	1364
Black bear	1352	955	397	1413
Leopard cat	1974	1021	953	1982
Red fox	1862	983	879	1873
Weasel	1269	728	541	1296
Wild boar	1875	1215	660	2007
Total	9641	5823	3818	9935

**Table 2:** Comparison of the improved backbone network model with the ResNet-18 backbone network model on the dataset.

Model	Parameter	${\mathrm{mAP}}_{50}$ %	*mAP*_50–95_ %
ResNet-18 backbone network (RT-DETR decoder)	15.4 M	0.949	0.772
Improved backbone network (RT-DETR decoder)	8.2 M	0.959	0.78

Both the ResNet-18 backbone network and the improved backbone network do not include MCCA and DFAM.

**Table 3:** Ablation experiments of the three key components on the dataset constructed in this paper.

Improved Backbone Network	MCCA	DFAM	P %	R %	${\mathrm{mAP}}_{50}$ %	*mAP*_50–95_ %
ResNet-18 backbone network (RT-DETR decoder)			0.951	0.931	0.949	0.772
✓			0.957	0.94	0.959	0.78
✓	✓		0.964	0.95	0.967	0.794
✓		✓	0.969	0.937	0.956	0.795
✓	✓	✓	0.98	0.964	0.978	0.819

**Table 4:** Comparison of MDA-DETR with 6 state-of-the-art methods.

Model	P %	R %	${\mathrm{mAP}}_{50}$ %	*mAP*_50–95_ %	Parameter	FPS
RT-DETR-r18 [11]	0.958	0.934	0.965	0.806	21 M	52.6
yolov8n [42]	0.943	0.926	0.972	0.798	3.2 M	76.9
yolov9-C [14]	0.971	0.958	0.975	0.809	25.5 M	71.5
DETR [10]	0.935	0.921	0.948	0.741	41 M	57.4
Deformable-detr [18]	0.957	0.927	0.967	0.757	40 M	59.8
DCA-yolov8 [43]	0.947	0.938	0.973	0.801	11.5 M	102
MDA-DETR (ours)	0.98	0.964	0.978	0.819	18.4 M	54.5

## Data Availability

This study uses the publicly available Northeast China Tiger and Leopard National Park dataset, which can be downloaded at https://github.com/myyyyw/NTLNP (accessed on 9 March 2023).
